# Development of Calvarial Bone Osteomyelitis in a Noonan Syndrome Patient Following Fronto-Orbital Advancement of Craniosynostosis: A Novel Technique of Treatment Using Povidone and Hydrogen Peroxide Solutions

**DOI:** 10.7759/cureus.52719

**Published:** 2024-01-22

**Authors:** Ikhlass S Altweijri, Taghreed R Alhumsi, Abdullah S Alsahli, Faisal A Al Jabr, Dana W Alkuwaity

**Affiliations:** 1 Pediatric Neurosurgery, Neurosurgery Division, King Khalid University Hospital, Riyadh, SAU; 2 Plastic Surgery Department, The Clinics, Riyadh, SAU; 3 Neurosurgery Department, Medical Services of Ministry, Security Forces Hospital, Riyadh, SAU; 4 Plastic Surgery Department, King Fahad Hospital Hofuf, Al-Hofuf, SAU; 5 Surgery Department, King Faisal University, Al-Hofuf, SAU

**Keywords:** osteomyelitis, fronto-orbital advancement, novel technique, noonan syndrome, craniosynostosis

## Abstract

This is a case of a pediatric patient with Noonan syndrome (NS) and craniosynostosis who developed calvarial bone osteomyelitis following corrective surgery. Despite complications, such as postoperative bleeding and infections, including osteomyelitis, multidisciplinary management strategies were employed, including antibiotics, debridement, and novel use of hydrogen peroxide and povidone solutions due to bone thinning. The discussion highlights challenges in managing syndromic craniosynostosis, emphasizing the importance of tailored approaches and prophylactic antibiotics. The innovative treatment approach using hydrogen peroxide and povidone presents a potential alternative for bone infections and osteomyelitis post-cranial reconstruction, offering insights for future management strategies. Lessons learned regarding infection rates and novel treatment modalities contribute to evolving approaches in managing complications in syndromic craniosynostosis.

## Introduction

Craniosynostosis is a congenital anomaly in which there is a premature fusion of one or more cranial sutures. Treatment is indicated to prevent or treat elevated intracranial pressure (ICP), to aid in normal social interaction, to prevent the decrease in the intracranial volume, and to restrict the brain growth causing elevated ICP [1,2]. Several surgeries can be done during the first year of life, such as posterior cranial vault remodeling, posterior vault reconstruction with distraction osteogenesis, and fronto-orbital advancement (FOA) [2]. Complications following surgery include bleeding, venous air embolism, cerebrospinal fluid leak, osteolysis, and infection. Infections do occur post-operatively, reaching up to 8%, but their occurrence can be life-threatening. Calvarial bone osteomyelitis is a complicated problem and is challenging for reconstruction [1,3,4,5]. Given that this is the first report from Saudi Arabia and the necessity of neurosurgeons and plastic surgeons to be aware of how to deal with such a condition, this study aims to present a new surgical approach to manage patients with Noonan syndrome (NS) who developed calvarial bone osteomyelitis and surgical site infection after craniosynostosis correction.

## Case presentation

The patient was an 18-month-old boy with NS, which was confirmed by genetic testing. Craniosynostosis was diagnosed by a computed tomography (CT) scan of the head including skull 3D reformats, showing polyostotic craniosynostosis with premature closure of metopic, sagittal, right squamosal, and caudal right coronal sutures. The anterior and posterior fontanelles were closed, and crowding of the brain parenchyma was noted with bilateral tonsillar herniation. 

After one day of admission, the patient underwent FOA and cranio-orbital reshaping under general anesthesia. During the operation, he had episodes of hypothermia and oozing, and the estimated blood loss was 500 cc. After the procedure finished with the dressing being applied, there was an accidental extubation of the endotracheal tube, and anesthesia had to re-intubate the patient with the size of 4.5 tubes. During re-intubation, one staff was leaning on the patient’s head from the left side to the right orbit, and then the team and nurses were instructed immediately and clearly not to apply pressure on the patient’s forehead and reconstructed area. The patient was transferred then to the pediatric intensive care unit in stable condition after re-intubation. The post-operative plan was to start three doses of dexamethasone, administer intravenous (IV) cefazolin, keep the patient’s head elevated at 45 degrees, put no pressure on the frontal area of the skull, keep the drain under negative pressure, keep the dressing dry and clean, be on ventilation and pain killer, and order complete blood count (CBC) and CT of the brain and skull 3D in the same day. CT scan of the head was done later in the day, and it showed a fracture of the supra-orbital bar and lateral left orbital rim; then, the patient was taken to the operation room (OR) as category 1 to avoid intra-orbital injury due to fractured segments. Intraoperative findings showed severely bent plates and fractures along the supra-orbital bar and left orbital rim. All fractures were repaired and fixed with bur holes and running 2.0 PDS. No major bleeding was encountered and closure was done again.

On day one post-operation, a CT scan showed a global decrease in the gray-to-white matter differentiation with sulcal effacement and reduced ventricular size, highly suspicious for brain edematous changes. Bifrontal extra-axial collections showed improvement in the form of decreased air and maximum axial dimensions of 4 mm compared to 7 mm pre-operatively. Descending trans-tentorial herniation was noted. The patient was vitally stable, intubated, and ventilated, comfortable on sedation, with pupils 1-2 mm sluggish reaction but equal. The infectious disease unit was consulted given his new CT findings and recommended to discontinue cefazolin and no need to start antibiotics as the patient was afebrile and his CBC findings were normal. Furthermore, the patient was kept intubated and fully sedated with the head of bed elevation at 30 degrees. Three percent hypertonic saline was started to keep sodium 145-155 and kept on dexamethasone and Jackson-Pratt drain. 

On day two post-operation, the patient was hemodynamically stable and the dressing was dry and clean, with a drain output of 100 mL Haemoserous. CT scan of the brain showed preserved grey-white matter differentiation with interval improvement of the previously global sulcal and ventricular effacement, with tonsillar herniation noted with extra-axial right frontal collection measuring 4 cm.

After 26 days of the operation, the patient was discharged in good condition and was followed up regularly in the clinic with instructions for frequent dressing changes. 

After 15 days from the discharge, the patient was presented to the clinic with redness along the wound, associated with a 3 cm gap, pus discharge, and spiking fever. CT scan of the brain with contrast was done on the same day showing an increase in the previously noted extra-axial right frontal collection from 0.4 cm to 1 cm, with meningeal enhancement post-contrast, with tonsillar herniation noted. One day later, the patient underwent wound debridement under general anesthesia, and multi-compartmental abscesses were found. MRI of the brain was done post-operatively on the same day showing swelling and abnormal signal intensity and enhancement seen along the left temporalis muscle. The size of the previously seen bilateral epidural collections underneath the surgical site and over the frontal convexities was similar; however, they were now largely replaced by air, especially on the right side together with the presence of air-fluid levels. No evidence of hydrocephalus or diffusion restriction to suggest epidural abscess collection was found. Cerebellar tonsillar herniation was again noted. The plan for initial empirical antibiotics included meropenem, vancomycin, and amikacin and to keep the dressing intact with the drain being on negative pressure. Four days post-wound debridement, tissue culture showed a heavy growth of *Pseudomonas aeruginosa*, moderate growth of *Klebsiella pneumoniae*, and light growth of *Staphylococcus aureus*. The patient was started on cefepime, budesonide, and topical fusidic acid ointment, and meropenem was stopped. Amikacin was discontinued on day 10, while vancomycin and cefepime were continued without changes.

Three weeks post-wound debridement, MRI of the brain was done and showed an interval progression of the epidural collections seen over the frontal convexities and vertex bilaterally with diffuse multiple areas of diffusion restriction, highly suspicious for epidural abscesses, predominantly over the left frontal region and vertex with smaller ones over the right frontotemporal region. Suspected herniation of the right frontal lobe through a dural defect into the epidural space was noted.

**Figure 1:**
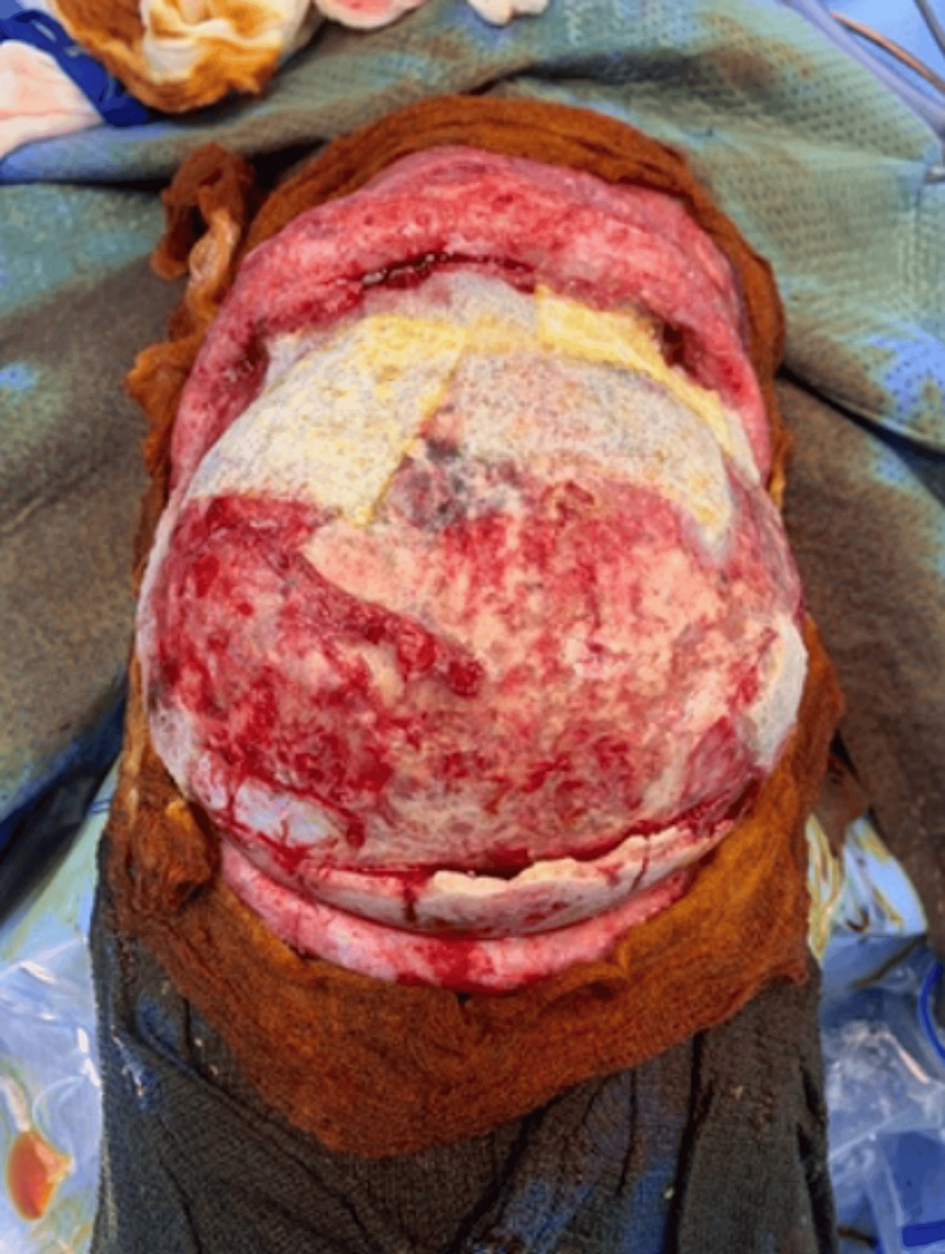
Superior-posterior view of the calvarial bone after three cycles of immersion with hydrogen peroxide and povidone.

**Figure 2:**
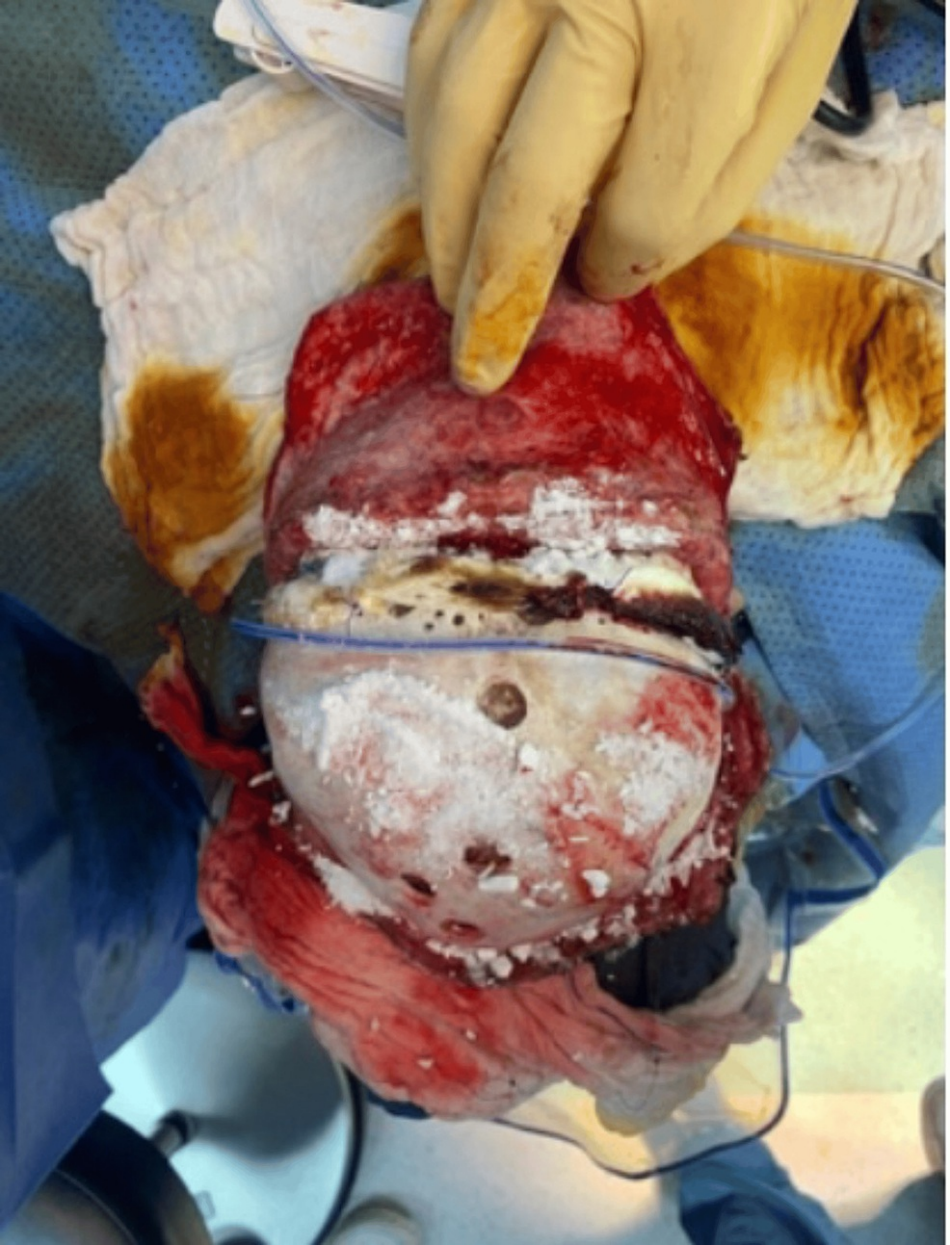
Superior view of the calvarial bone after three cycles of immersion with hydrogen peroxide and povidone.

**Figure 3:**
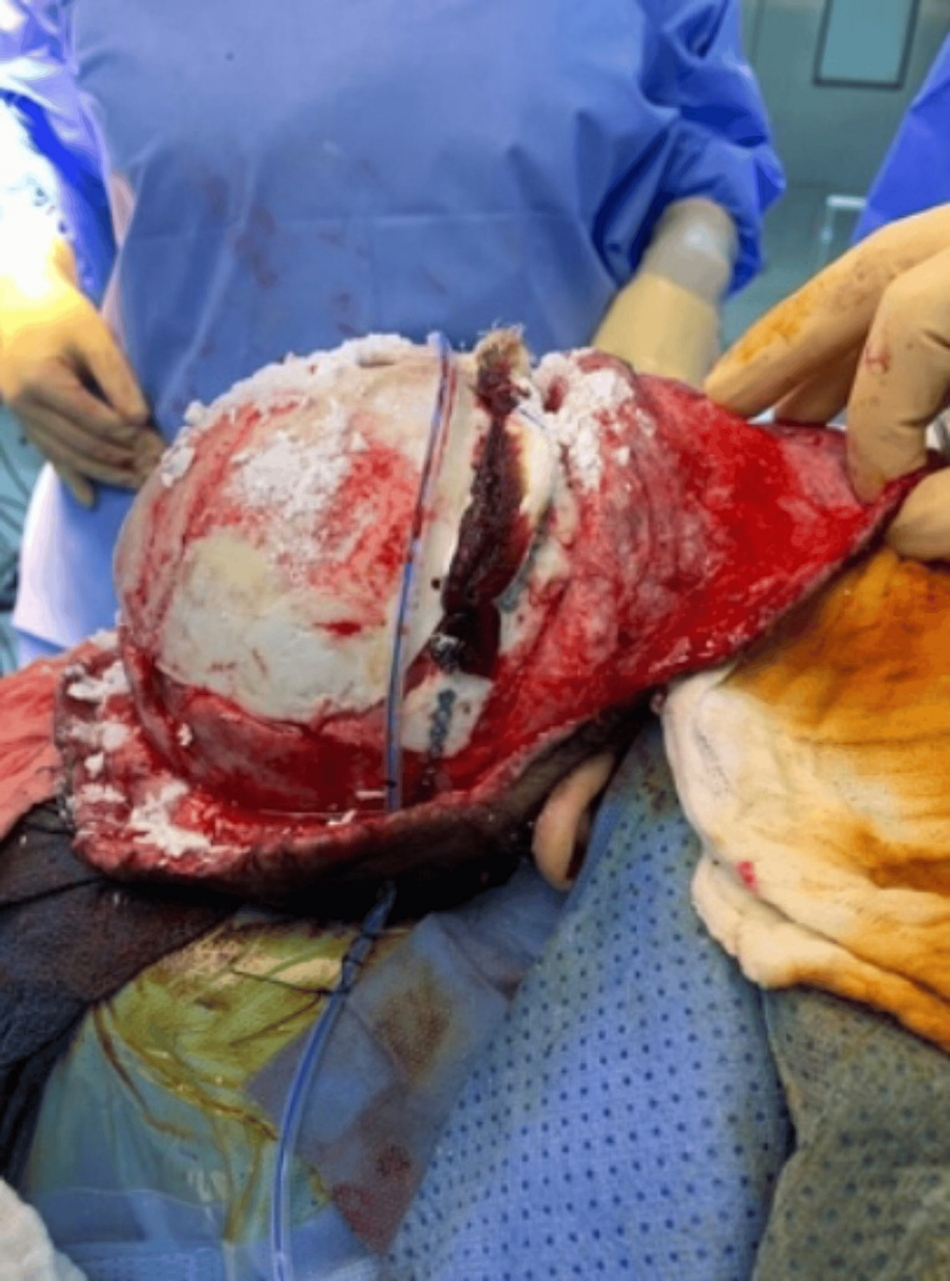
Right lateral view of the calvarial bone after three cycles of immersion with hydrogen peroxide and povidone.

**Figure 4:**
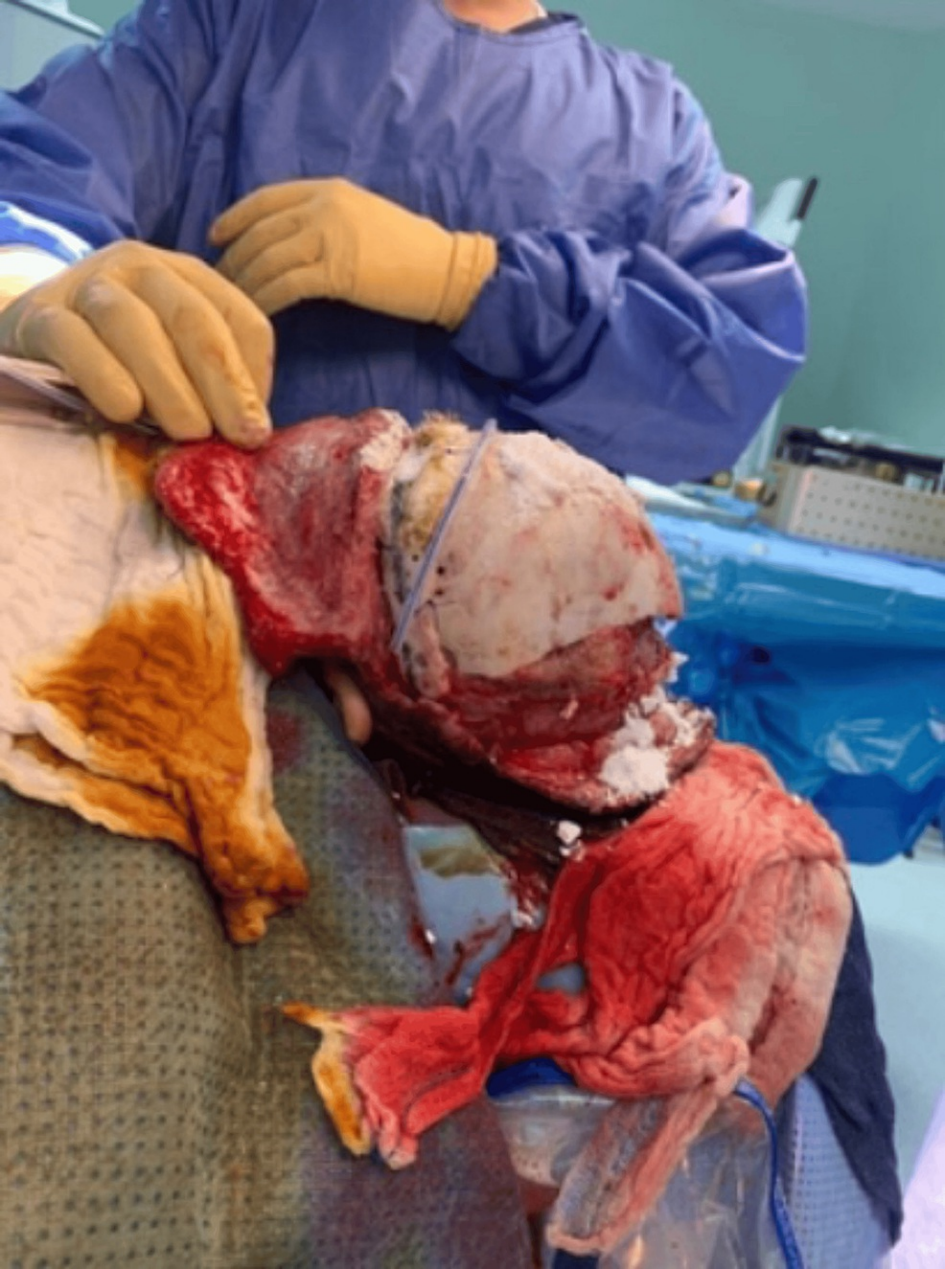
Left lateral view of the calvarial bone after three cycles of immersion with hydrogen peroxide and povidone.

The patient then underwent calvarial bone debridement and epidural abscess evacuation under general anesthesia, with an estimated blood loss of 100 mL. During the operation, epidural abscess, dural tear, and right frontal lobe herniation were found. The calvaria bone was infected, and bony debridement was done until the bone became very thin. A new approach was used during the operation for the infected bone, in which the calvarial bone was immersed in povidone solution for 20 minutes and then immersed into hydrogen peroxide for 20 minutes. This was repeatedly done three times, and then the bone became healthy and clean, as shown in Figures 1-4.

On day one post-operatively, the patient was doing well, vitally stable, and intubated on minimal sedation, moved all limbs, and opened eyes spontaneously. Urine looked cloudy, lumbar drains were 6 mL, and J-VAC was 95 mL. CT scan of the skull 3D reformats was done on the same day and showed interval reduction in the bifrontal convexity subdural collection with persistent pneumocephalus. On day three post-operatively, the lumbar drain was removed, and JVAC was 20 mL.

Two weeks following the operation, the patient developed bleeding from the surgical site and the hematology department was consulted. There were no other bleeding sites from the body, and there was no family history of bleeding disorders. The recommended plan was to start fresh frozen plasma (FFP) 10 mL/Kg daily for five days and vitamin K 3 mg daily for five days, to keep hemoglobin above 9 mg/dL, keep platelet count above 100,000, and monitor daily CBC, coagulation profile with fibrinogen level, factor IX, and vWF.

Five weeks following the operation, there was minimal fresh blood in the external ventricular drain (EVD) and no other bleeding sites from the body. The hemoglobin level was 10 mg/dL, platelet count was 447,000, white blood cell count was 7,800 cells/uL, coagulation profile was normal, and factor IX level was 56 units/dL. The vitamin K and FFP were discontinued, and in case of significant bleeding, the patient can be given activated factor XII 90 mcg/Kg intravenously. 

At the same time, the immunology team was consulted about the possibility of immunodeficiency. The immunoglobulin levels IgM, IgG, IgA, and IgE were ordered, and all were within the normal reference range.

The lymphocytes and flow cytometry results are shown in Table 1.

**Table 1:** Lymphocytes and flow cytometry results with their reference values.

Detail	Value	Flags	Normal Range
CD16/CD56%	13.67%	High	5.00-10.00
CD16/CD56 Abs	0.285 cells/uLx10^-3		0.200-0.600
CD19 %	54.20%	High	20.00-27.00
CD19 Abs	1.132 cells/uLx10^-3		0.700-1.600
CD3 %	29.21%	Low	61.00-70.00
CD3 Abs	0.580 cells/uLx10^-3	Low	2.200-4.100
CD4 %	18.13%	Low	36.00-44.00
CD4 Abs	0.341 cells/uLx10^-3	Low	1.400-2.800
CD8 %	10.74%	Low	20.00-30.00
CD8 Abs	0.202 cells/uLx10^-3	Low	0.800-1.800
CD4:CD8+ Ratio	1.69		1.30-2.100

## Discussion

NS is a pleomorphic autosomal dominant disorder, with an incidence being approximately one per 1,000-2,500 live births worldwide [6,7]. Patients can present with short stature, low set of ears, congenital heart disorders, cryptorchidism, and fetal macrosomia. Moreover, they can present with easy bruising and bleeding, most commonly from factor IX deficiency [7]. Although there is no clear evidence for an association between NS and immunodeficiency, two studies found a co-existence between NS and recurrent infections [8,9]. While NS has various phenotypic features, craniosynostosis is not a recognized feature and is not common among these patients [10]. In our case, the patient developed bleeding from the surgical sites two weeks following the operation for treating calvarial bone osteomyelitis, which was treated with FFP and vitamin K and following laboratory results regularly. The patient was improved thereafter, and all laboratory results were within the normal reference ranges. Moreover, one of the issues that faced us was the recurrent infection and the low CD3, low CD4, and low CD8 antibodies. However, the immunoglobulin levels were all within normal ranges.

Infectious complications following craniosynostosis correction occur with a percentage being around 0-8%, but the rate and severity increase for patients who are syndromic and undergoing re-operation for secondary reconstruction [3,11,12]. Infections can be superficial soft tissue infection, infected hardware, infected shunt, subgleal infection, intracranial infection, and osteomyelitis [3]. Our patient has NS and polyostotic craniosynostosis that underwent FOA followed by repair of bilateral broken supraorbital plates. The patient later was complicated by SSI and calvarial osteomyelitis. Calvarial osteomyelitis requires aggressive and early treatment with culture-guided IV antibiotics and surgery [4,13]. Surgical options for treating osteomyelitis are various and include the following: debridement with the removal of infected bone and tissue, removal of any foreign objects, draining the infected area, autoclaving, and amputation. One study was conducted for a non-syndromic patient who developed calvarial bone osteomyelitis following cranial vault reconstruction for craniosynostosis. Their technique was to send the autologous bone flap for bone processing and storage, in which ultrasonication of the bone in solutions of detergent, hydrogen peroxide, gentamicin, and isopropanol was done. Then, the bone was packaged and subjected to a low-dose gamma irradiation; after that, the bone was stored in an ultralow freezer until needed for re-implantation [4]. In our case, we are proposing a new technique as a useful option for treating calvarial bone osteomyelitis following pediatric calvarial reconstruction. In this approach, as presented in Figures 1-4, the calvarial bone was infected, and debridement was done until the bone thickness became very thin. Thereafter, the bone was soaked in a povidone solution and hydrogen peroxide solution, 20 minutes for each. This was repeated for three cycles until the calvaria became healthy, clean, and out of infection, and then the bone was replaced again. There was no need for storage or bone processing. One of the issues we faced was that during debridement, the calvarial bone became very thin, so we were not able to do autoclaving, and the patient was pediatric.

In addition, since the surgical treatment for craniosynostosis is usually done during infancy, pre- and post-operative antibiotic administration is of utmost importance to avoid the possibility of wound infection, osteomyelitis, and poor wound healing [12]. A systematic review of the use of pre- and post-operative prophylactic antibiotics used in craniosynostosis-correcting procedures found that limiting the administration to a preoperative single dose or up to 24 hours after the operation can be clinically applied [12]. In the case of a suspected osteomyelitis, empirical broad-spectrum antibiotics should be started initially as soon as the bone biopsies have been obtained. Thereafter, selective antibiotics are given according to all results of isolates and/or suspected microorganisms [14,15]. In regard to our patient, he received one dose of cefazolin pre-operatively and continued for 24 hours post-operatively for the craniosynostosis correcting procedure. Once he was suspected to have calvarial bone osteomyelitis, the initial empirical antibiotics that were started were meropenem, vancomycin, and amikacin. Meropenem is considered a good option in these cases due to its broad-spectrum coverage and showed good positive outcomes from the previous literature [16]. Tissue culture showed heavy growth of *Pseudomonas aeruginosa,* moderate growth of *Klebsiella pneumoniae*, and light growth of *Staphylococcus aureus*. The patient then started cefepime, budesonide, and topical fusidic acid, and meropenem was stopped. Amikacin was discontinued on day 10, while vancomycin and cefepime were continued the same.

During the treatment of our patient, several lessons were learned. First, the infectious rate following craniosynostosis can increase significantly since the patient is syndromic, has polyostotic craniosynostosis, and has undergone a secondary correcting procedure. Also, the cefazolin used as a sole antibiotic preoperatively and continued for 24 hours post-operatively was sufficient to protect against infections for the first correcting procedure. Moreover, the novel method we have used during the operative treatment of calvarial bone osteomyelitis with the use of hydrogen peroxide and povidone cycles can be used as a novel method. Lastly, after the bone became very thin during debridement, autoclaving was not an appropriate option for the patient.

## Conclusions

Calvarial bone osteomyelitis following craniosynostosis correction is challenging, especially in NS patients. In this case, the patient developed osteomyelitis of the calvarial bone following a craniosynostosis corrective procedure, in which he was started on empirical antibiotics and debridement was done resulting in a very thin and delicate bone. Calvarial bone debridement followed by immersing the bone in povidone and hydrogen peroxide solutions for 20 minutes, repeated for three cycles, eradicated the bony infection without the need for bone processing or storage.
